# Assessing the Preferences for Criteria in Multi-Criteria Decision Analysis in Treatments for Rare Diseases

**DOI:** 10.3389/fpubh.2020.00162

**Published:** 2020-05-08

**Authors:** Carina Schey, Maarten Jacobus Postma, Paul F. M. Krabbe, Olekdandr Topachevskyi, Andrew Volovyk, Mark Connolly

**Affiliations:** ^1^Global Market Access Solutions, St-Prex, Switzerland; ^2^Unit of Pharmacotherapy, Epidemiology & Economics, Department of Pharmacy, University of Groningen, Groningen, Netherlands; ^3^Department of Health Sciences, University Medical Center Groningen (UMCG), University of Groningen, Groningen, Netherlands; ^4^Department of Economics, Econometrics & Finance, Faculty of Economics and Business, University of Groningen, Groningen, Netherlands; ^5^Department of Epidemiology, University of Groningen, University Medical Center Groningen (UMCG), Groningen, Netherlands; ^6^Department of Health Economics, Digital Health Outcomes LLC, Kyiv, Ukraine

**Keywords:** multi-criteria decision analysis, interactive tool, focus group, weights, preferences

## Abstract

**Background:** Increasingly, multi-criteria decision analysis has gained importance as a method by which to assess the value of orphan drugs. However, very little attention has been given to the weight (relative preferences) of the individual criteria used in a framework.

**Aims:** This study sought to gain an understanding of the preferential weights that should be allocated in a multi-criteria decision analysis framework for orphan drugs, from a multi-stakeholder perspective.

**Method:** Using key MCDA criteria for orphan drugs reported in the literature, we developed an interactive web-based survey tool to capture preferences for different criteria from a general stakeholder sample who were requested to assign weights from a reimbursement perspective. Each criterion could be assigned a weight on a sliding scale from 0 to 100% as long as the sum of all the criteria was 100%. We subsequently used the interactive tool with an expert focus group, followed up with a group discussion regarding each criterion and their perspectives on the weight that each criterion should be allocated when assessing an orphan drug. The expert focus group participants were then able to adjust their weights, if the group discussion had changed their perspectives.

**Results:** The interactive tool was completed by 120 general stakeholder sample from a wide range of countries and professional backgrounds and an expert focus group of ten members. The results showed the differences in perspectives on the importance of criteria. Both groups considered *Treatment efficacy* to be the most important criterion. The general stakeholder sample weighted *Treatment safety* at 12.03% compared to the expert focus group's average of 20%. The results also demonstrated the value of the group discussion, which provided additional insights into the perspectives on the importance of criteria in assessing orphan drugs.

**Conclusion:** This study aimed to contribute to the important aspect of preferences for different criteria in MCDA. This study sheds light on the important aspect of the preferences of the different criteria. All respondents agreed on the relative importance of *Treatment efficacy* and *Treatment safety*, criteria that are captured in conventional cost-effectiveness studies, but they also expressed the view that in addition to those, several disease-related and drug-related criteria should be included in MCDA frameworks for assessing orphan drugs.

## Background

Decision-making in healthcare is usually a process whereby different alternatives are identified and compared to find the best solution based on multiple factors that address the decision-makers' and the organization's expectations (1). With the launching of new and in some cases, innovative treatments such as gene therapy for rare diseases, it's likely that the increasing demands for the reimbursement of such treatments will raise added concerns on the long-term affordability, especially of expensive treatments (2, 3). In order to mitigate the uncertainty of the health benefits of treatments that have been trialed for relatively short periods of time, healthcare systems might have to adopt novel ways of reimbursement decision-making, in addition to cost-effectiveness studies, which is the most commonly used way of assessing the value-add of a new treatment (4, 5). Several decision-making approaches have been developed in healthcare, such as the three-talk model (6) that focuses on shared decision-making in clinical practice, and the use of health outcomes and economic analyses (7). In particular, since the introduction of the Orphan Drug Regulation in Europe in 2000 (8), healthcare systems and reimbursement bodies have had to consider new ways of assessing the value-add of rare disease treatments. Methodologies have ranged from cost-effectiveness models (5, 9), to budget impact models (10) and other tools such as multi-criteria decision analysis (MCDA) (11).

While the European Medicines Agency (EMA) grants the marketing authorization for treatments in Europe (12), the reimbursement decisions are made a local country level (13, 14). Despite the introduction of the Mechanism of Coordinated Access to orphan medicinal products (MoCA) (15), and the commitment by member states to collaborate on improving access to treatments for rare diseases (16), access to orphan drugs across Europe remains variable due to different reimbursement decisions in each country (14, 17). In England, for example, less than 50% of centrally authorized orphan drugs are funded by the National Health Service, with one-third of these recommended by the National Institute for Health and Care Excellence (NICE) (14). The issue of access to orphan drugs might further be complicated by the difference in pricing (18) and pricing negotiations from one country to another, and the metrics by which payers adjudicate the added value of the orphan drug (19). In light of these complex issues, and the increase in expensive orphan drugs (20, 21) different models are being proposed to increase the confidence in the evidence for reimbursement from pre-launch all the way through to post-launch activities. Furthermore, some alternative reimbursement models include patient access schemes (22), reference pricing in pricing negotiations, “cost plus” pricing imposed by the health technology assessment body (23), discount pricing (24) managed entry agreements or managed access agreements (25) and rebate schemes (26), (27). Ultimately, payers are faced with an increasing need to use robust measures with which to assess if new orphan drugs demonstrate value for money, while considering the real clinical benefits and risk of adverse events for the patients, while not wasting medical resources (28).

Recently, MCDA has gained increasing attention in reimbursement decisions for orphan drugs (29, 30) as an alternative to cost-effectiveness. The practice of MCDA in decision-making processes has been used extensively in many industries such as the aviation industry (31) and energy and environmental industries (32) for many years. This particular interest in MCDA is due to the belief that the traditional cost-effectiveness approach used to assess the value of orphan drugs is not robust enough to capture all the multi-dimensional factors that inform on the real benefits of the treatment under review (33, 34). Some of the earliest use of MCDA in healthcare include a study in 1989 to review options in pyelonephritis (35) and for haemoglobinopathies (36). Subsequent use of MCDA in healthcare has been intermittent. To support the development of MCDA for orphan drugs and decision-making by health technology assessment (HTA) bodies and payers, The International Society for Pharmacoeconomics and Outcomes Research (ISPOR) Taskforce in multi-criteria decision analysis published recommendations on MCDA (37, 38). However, the ISPOR Taskforce reports are not payer-centric and don't provide insights on how to address the criterion of cost of orphan drugs.

The principle of MCDA is that it provides a matrix or framework whereby multiple factors that describe the disease and treatment under review can be arranged and assessed. The MCDA framework includes the different drug options, the criteria which measure the outcomes of the drugs being assessed, the scaling system by which criteria are measured (allocated a score) and the weights ascribed to the different criteria (38–40). The choice of criteria is open to interpretation depending on the decision question, although there seems to be a common list of criteria that are considered appropriate for assessing orphan drugs using MCDA, as described in the literature (34, 41, 42). Preferences or weights, that describe the relative importance of the criteria against which the different treatments are compared, can then be ascribed to each criterion. Finally, a total “drug score” can be aggregated and used comparatively against other drugs in decision-making and resource allocation (37, 43). Thereby, the MCDA framework provides the opportunity to compare qualitative and quantitative outcomes (44). There are various methods in which to apply MCDA, with the most commonly used ones being the multi-attribute utility theory (MAUT), Analytic Hierarchy Process (AHP), and Outranking (45).

MCDA is being used in some healthcare systems to support the allocation of resources. In England, the Advisory Group for National Specialized Services (AGNSS) was the first health technology assessment body to adopt MCDA to assess orphan drugs (46) using a framework based on efficacy, societal value of the new treatment, the cost of the new treatment, and impact of the new treatment on service delivery (47). AGNSS was subsequently taken over by the Highly Specialized Technologies (HST) group for NICE, who also use the MCDA approach (48). In Spain, the Spanish Agency for Medicines and Health Products developed a MCDA framework based on the EVIDEM framework (49) and similarly in Catalonia (50). The region of Lombardia in Italy has adopted a MCDA approach to regulate the introduction of new health technologies. Their MCDA is based on the EVIDEM framework. The introduction of this formal MCDA model stemmed from the desire to balance goals of continuous innovation with the needs of steady cost containment, and to instill uniformity and transparency in a process that may be highly complex. While subjectivity cannot be completely removed, the framework seeks to minimize discretion in decision making and to produce decisions perceived as legitimate by all the stakeholders (51).

Interestingly, the very earliest reference to MCDA seems to be in a letter that Benjamin Franklin (one of the Founding Fathers of the USA) wrote to an acquaintance in 1772. Benjamin Franklin explained that when faced with multiple factors that influence a decision, it is worth assessing the problem by dividing a page into two columns labeled *Pros* and *Cons* under which the decision-maker can list all the necessary factors. He further described his method by saying “When I have thus got them all together in one view, I endeavor to estimate their respective weights; and where I find two, one on each side, that seem equal, I strike them both out. If I find a reason pro equal to two reasons con, I strike out the three. If I judge some two reasons con, equal to some three reasons pro, I strike out the five; and thus proceeding I find at length where the balance lies; and if, after a day or two of further consideration, nothing new that is of importance occurs on either side, I come to a determination accordingly” (52).

Benjamin Franklin's letter alludes to a key point in the use of MCDA, and particularly in assessing orphan drugs—namely the use of weights, or preferences of the different criteria, thereby implying that not all criteria should carry the same importance and that the MCDA model should reflect the weighted preferences. In assessing orphan drugs using MCDA, it could therefore be postulated that *Disease severity* might be more important than *Disease rarity*, for example. The weighting is used when aggregating all the criteria to establish the overall “value” (score) of the drug, and the weighting score allocated to a criterion is independent of individual score given to each criterion. For example, the *Treatment convenience* of a drug might be scored as “high” (meaning that the drug offers easy administration with low associated costs), but the criterion is not considered important by the adjudicating panel, and is therefore given a low weight (preference) in aggregating all the criteria scores for each drug.

While ample research has been performed in recent years on the suitable criteria for inclusion in a MCDA model for orphan drugs (42, 50, 53), less research seems to have been done on the weights or preferences that should be applied to the different criteria. The weighting scales used in research range from 5-point scales (50, 54), to 10-point scales (55) and several studies mention using a 100-point scale (34, 41, 56). A pilot study by Reddy et al. included a group of eight participants (plus the facilitator) to select the criteria that should be considered for a MCDA framework for several public health preventative programmes. They adopted an AHP approach to weight each criterion, thereby obtaining a total score for each under discussion (57). Although this pilot study focused on the choice of topics with which to assess which criteria for public health programmes rather than for rare disease treatments, it highlights the benefits and some of the potential pitfalls of using AHP and MCDA.

The aim of this study was to ascertain the preferences for different criteria from many participants, by comparing the results gathered in a large respondent pool with those from a focus group of experts. The target audience is anyone interested in assessing if MCDA has a role to play in reimbursement decisions for orphan drugs. The authors hope that the research would help further increase the dialogue on the use of alternative methods to assess the value of orphan drugs in addition to cost-effectiveness. Intentionally, the authors did not include a discussion on orphan drug prices, as the key aim was to better understand the weight preferences for all other criteria that might be included in a MCDA framework. In the follow-up study that is planned using a far larger group of respondents from many different countries, the orphan drug prices will be prioritized for debate.

## Method

Using key MCDA criteria for orphan drugs reported in the literature (34, 41, 42, 58), we developed a web-based interactive tool (59) to capture preferences for different criteria from a general stakeholder sample attending medical, healthcare or health economic conferences. The criteria were divided into 4 major categories: Disease Burden, Product Development, Clinical Impact and Economic Impact. The tool was designed so that respondents could allocate any weight to the criteria by means of a sliding scale up to a total of 100% and allowing for the inclusion of as many of the criteria as the respondent wanted. i.e., not all criteria had to be allocated a weight. Respondents were asked to use the tool based on what they deemed important in reimbursement decisions. The tool invited respondents to select their field of expertise (e.g., academia, industry) or affiliation (patient representative organization) and the country in which they worked. Once the respondent had completed their weighting, a comparative output showed by how much their inputs deviated from the existing sample average, both as an overall average as well as a diagrammatic representation for each criterion.

We subsequently conducted an expert focus group to gather additional insights from different perspectives on the weights that could be allocated to different criteria when MCDA is used to assess orphan drugs. To this end, we recruited a panel of ten people, all based in Switzerland, comprised of two payers (one each from the state and private insurance sectors), two clinicians specialized in rare diseases (different diseases), two clinical Pharmacists (both working in large teaching hospitals), two health economists (one each from academia and private health insurance), and two patient representatives from different rare disease organizations. Panel recruitment invitations were distributed by personal contact with each participant, with explanations of the study outline, objectives and detailed descriptive information about each criterion included in the tool (summarized in Table 1).

**Table 1:** Summarized descriptions of criteria provided to the expert focus group.

Treatment efficacy	The degree of improvement of the standard outcome by which a drug's efficacy is measured against the most likely comparator in a specific disease.
Disease severity	An attribute that reflects the severity of: •Disease-related mortality •How symptomatic the disease is •Mental status (anxiety/depression) •*Physical implications &/disability*
Unmet need	A criterion that reflects: •Number of treatment alternatives •Benefit from the alternative treatment
Level of research undertaken	A criterion that considers: •Level of trials undertaken (e.g., phase 2 vs. phase 3) •Trial duration •Size of the trial: • Number of study centers • Number of patients included
Innovation	Based on a description of the ASMR system used by the HAS in France
Dynamic efficiency (R&D spillover)	Where the research and development costs for one indication leads to discovery of the drug's value in a second indication that does not require the same level of extensive R&D investment
Treatment safety	A criterion that summarizes the: •Serious adverse events in clinical trials (30%) •Treatment discontinuation due to adverse events (30%) •Treatment-related mortality (40%)
Treatment convenience	•Route of administration •Frequency of administration •Time to administer treatment •Duration of treatment (weeks, months, years)
Treatment follow-up measures	•Test complexity •Test frequency
Budget impact/Affordability	The local healthcare economy's perception of how much a new treatment will impact on the budget
Broader economic consequences	Any additional economic benefits that a treatment offers. E.g. being able to work, reduced absenteeism from school &/ work
Value for money (cost-effectiveness)	The criterion captures an understanding of how resources are transformed into valued health system outputs Smith, who, measuring value for money in healthcare, concepts and tools
Disease rarity	Based on levels of disease prevalence with a guideline of: •0.01 to 5 per 100,000 population •5.1 to 66 per 100,000 •67 to 100 per 100,000 •101 to 200 per 100,000 •201 to 300 per 100,000 •> 301 per 100,000

We conducted the focus group session according to published focus group methodology (60) in a face-to-face group session held at the hospital in Lausanne, Switzerland. The session was run in English and all participants were fluent English speakers. We initiated the session by describing each criterion and demonstrating how the interactive tool worked. The focus group participants were then invited to each use the interactive tool and to complete the weighting exercise from the perspective of their employment role rather than from a societal perspective. In contrast to the larger study, the focus group participants were asked to provide reasoning for their choices. Following a group discussion of each criterion, the participants had the opportunity to change their weighting if they so desired.

## Results

### General Stakeholder Sample Weight Preference Data Collection

The interactive tool was completed by 120 respondents from a wide range of professional backgrounds and multiple countries. The country representation and professional affiliations of the large pool respondents are listed in Tables 2, 3, respectively. The largest representation of respondents were from the USA (13%), the Netherlands (12%), Belgium, and Germany (both 10%). The highest number of professional affiliations were Academia and Industry (Pharmaceutical/Medical devices/Diagnostics) at 27% and 23%, respectively.

**Table 2:** Countries of origin of the general stakeholder sample.

Afghanistan	1%
Albania	3%
Armenia	1%
Australia	3%
Belgium	10%
Bosnia & Herzegovina	1%
Brazil	2%
Canada	2%
China	1%
Cyprus	1%
Denmark	1%
Finland	1%
France	5%
Germany	10%
Greece	3%
Italy	1%
Latvia	1%
Lithuania	1%
Luxembourg	1%
Netherlands	12%
Romania	2%
Russian Federation	1%
Serbia	1%
Spain	2%
Sweden	1%
Switzerland	8%
United Kingdom	9%
Ukraine	8%
United States of America	13%

**Table 3:** Professional affiliations of the general stakeholder sample.

Industry/pharmaceutical/medical devices/diagnostic	23%
Academia	27%
Patient representative	10%
Health research/consulting	9%
Government/HTA/non-profit	9%
Clinical practice/hospital	7%
Managed care/pharmacy benefit management	4%
Patient	8%
Biotech	3%

**Figure 1:**
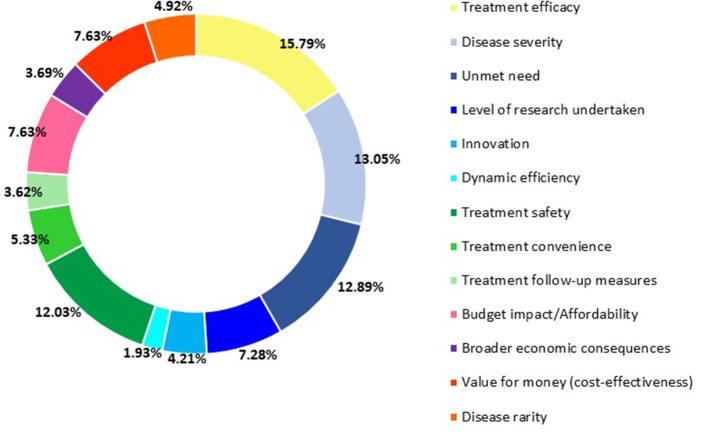
The average weight scores for the general stakeholder sample.

Figure 1 shows the average weights of the criteria for all respondents in the general stakeholder sample, which highlights that *Treatment efficacy* was considered the criterion that deserved the highest weight, followed closely by *Disease severity* and then *Unmet need*. The respondents scored *Dynamic efficiency (R&D spillover)* as the criterion with the least importance.

### Focus Group Weight Preference Data Collection and Discussion

Table 4 lists the weight preferences for each of the participants of the expert focus group session after the group discussion and having had the opportunity to change their choices if they so wished.

**Table 4:** Weight allocations after the focus group discussion for the expert focus group.

	**Criterion**	**CL 1**	**Cl 2**	**PH 1**	**PH 2**	**HE 1**	**HE 2**	**PY 1**	**PY 2**	**PR 1**	**PR 2**
Disease	Disease severity	11%	15%	11%	10%	10%	10%	5%	10%	10%	10%
	Disease rarity	0	0	0%	0	0	0%	0	0	0	0
	Unmet need	10%	11%	13%	5%	10%	10%	0	0	10%	10%
Product development	Level of research undertaken	10%	11%	15%	21%	8%	10%	10%	13%	15%	15%
	Innovation	0	0	0	4%	3%	5%	0	0%	0	5%
	Dynamic efficiency (R&D spill over)	0	0	0	0	2%	5%	0	0%	0	0
Clinical impact	Treatment safety	26%	17%	22%	18%	18%	20%	18%	15%	20%	23%
	Treatment efficacy	26%	25%	22%	20%	18%	20%	18%	19%	25%	20%
	Treatment convenience	8%	3%	0	0	8%	0	9%	0	5%	5%
	Treatment follow up measures	9%	6%	0	0	5%	0	3%	8%	5%	5%
Economic impact	Budget impact/affordability	0	0	17%	0	9%	17%	11.0%	13%	0	0
	Broader economic consequences	0	12%	0	0	9%	3%	11.0%	11%	10%	7%
	Value for money (cost-effectiveness)	0	0	0	22%	0	0	15%	11%	0	0

*Colored cells: Red for decrease in weight; green for increase in weight after the expert group discussion*.

*CL: Clinician*.

*PH: Pharmacist*.

*HE: Health Economist*.

*PY 1: Payer (state)*.

*PY 2: Payer (private insurance)*.

*PR: Patient representative*.

The numbers in red cells are those that were decreased and those in green cells were increased. Both the medical doctors and the patient representatives were pleased with their initial choices, while all the other respondents made some changes.

The discussion provided some useful insights herewith summarized according to each criterion. The general consensus was that *Disease severity* can have a significant negative impact on the health-related quality of life of patients, especially for those with progressive rare, genetic disease. Following the group discussion, a Pharmacist and a Health Economist each decreased their weight allocation from 20 and 18%, respectively, to 10%, while the Payers increased their respective weights from 0 to 5% and 10%. By contrast, *Disease rarity* was considered to not be a significant criterion, and further downplayed following discussion, as seen in the changes made by 2 members (Pharmacist & Health Economist). It was felt that in light of the Orphan Drug Regulation in Europe, the rarity of disease was already accounted for in the incentives made available to drug manufacturers. As with the general stakeholder sample, the focus group felt that *Treatment efficacy* was the criterion that deserved the most weight, with *Treatment safety* in very close second.

Across the group, 70% of the members agreed that *Unmet need*, which related to the availability of suitable treatment(s), was an important criterion scoring it a weight of an average of 10.57% whereas 30% of the group believed that the criterion should somehow be linked to the drug's efficacy and safety, implying that *Unmet need* alone was not important enough to warrant a high weight.

The initial weight bestowed upon *Level of research undertaken* ranged from 5% (Health Economist) to 21% (Pharmacist). In three cases, the weight was increased following the discussion (pharmacist, health economist & public sector payer) and the private-sector payer (private sector) reduced the weight slightly (14–13%). *Innovation* is a criterion that was not considered at all important and on average was given a weight of only 2%.

*Dynamic efficiency (R&D spillover)*, where the development of a drug for one indication leads to the drug being effective in a second indication or the advent of added benefits from the research and development of the drug, was viewed as an unimportant criterion even though a subsequent indication could influence the price, sales volume and reimbursement negotiations.

*Treatment convenience*, which relates to the route of administration and frequency of administration, was awarded a relatively low weight. Similarly, *Treatment follow-up measures* was considered a relatively unimportant criterion, although one of the clinicians pointed out the potential cost implications of a treatment that would require very regular costly monitoring.

The economic criteria of *Budget impact/Affordability, Broader economic consequences* and *Value for money (cost-effectiveness)* were awarded a wide range of weights with *Budget impact/Affordability* being perceived as the most important of the economic criteria. These criteria experienced only minor changes in weights after the discussion. Overall, the average weights were 7, 6, and 5% for *Budget impact/Affordability, Broader economic consequences* and *Value for money (cost-effectiveness)*, respectively. The average results are shown comparatively for both sets of respondents in Table 5, which highlights significant differences in the perception of the 2 groups (the large group vs the expert group), most notably for *Treatment efficacy* and *Treatment safety* that are both favoured by the expert focus group. The variations between the groups with respect to the economic criteria are not significant.

**Table 5:** Comparative weights between the two study groups.

**Criteria groups**	**Criteria**	**Large group**	**Expert Focus group**
		**(*n* = 120) (%)**	**(*n* = 10) (%)**
Disease burden	Disease severity	13.05	10
	Unmet need	12.89	8
	Disease rarity	4.92	0
Product development	Level of research undertaken	7.28	13
	Innovation	4.21	2
	Dynamic efficiency (R&D spillover)	1.93	1
Clinical impact	Treatment efficacy	15.79	21
	Treatment safety	12.03	20
	Treatment convenience	5.33	4
	Treatment follow-up measures	3.62	4
Economic impact	Budget impact/Affordability	7.63	7
	Broader economic consequences	3.69	6
	Value for money (cost-effectiveness)	7.63	5

## Discussion

This study identified the perspective of a large group of people (120 respondents) and an expert focus group via an interactive tool, on the suggested weights of commonly cited criteria suggested for use in assessing the value of orphan drugs by means of MCDA. Although both groups applied the highest weight to *Treatment efficacy*, the results show that the expert group favored the Clinical Impact criteria (*Treatment efficacy, Treatment safety, Treatment convenience, Treatment follow-up measures*), and the large group, who did not have the benefit of any discussions, weighted the Disease Burden criteria (*Disease severity, Unmet need, Disease rarity*) the most.

With each group of respondents basing their choices from a reimbursement perspective as indicated in the instructions, the results suggest that having the opportunity to discuss the criteria and their impact within a small, expert group gives additional insights of the importance, and therefore the weights, of some key criteria. As a result of the group discussion, three members increased the weight for *Level of research undertaken* on the basis that the more extensive the clinical research, the less the uncertainty of outcomes in the real clinical setting, and therefore the more the criterion should be weighted with a scale that describes different levels of research. These changes made to *Level of research undertaken* by the expert group, and difference between the average weights allocated to the criterion by both groups suggests that the interactive forum assisted weight allocation by respondents.

One of the main advantages of the group discussion is that it provides a confidential platform for participants to openly comment, explain, disagree, and share their views, perspectives and knowledge (61). Although the focus group session enabled the members to respond in their own words and to share opinions in a non-judgmental way, it was also a structured session aimed at allowing us to obtain information pertinent to the study. In the pilot performed by Sussex et al., they included stakeholders who worked in a pharmaceutical company (GlaxoSmithKline), EU clinical and health economics experts, and representatives of rare diseases patient groups in the EU. In our study, we excluded representation from the pharmaceutical industry sector to ensure that the members felt free to share their opinions. We included payers from the state-funded and private insurance sectors to identify potential differences in perspectives.

One of the points that raised debate among the participants was what they referred to as “double counting,” especially for *Unmet need*, which initially several members felt was already captured in *Disease severity*. However, as one of the clinicians described, a patient could present with a very severe disease but for which there are several treatments, such as pulmonary arterial hypertension, in which case the unmet need is not substantial compared to an equally severe disease for which there is no treatment. The participants suggested that another area at risk of “double counting” are the economic criteria, and that clear differentiation needs to be made between *Budget impact/Affordability, Broader economic consequences, and Value for money/cost-effectiveness*. In particular, the sub-categories for *Broader economic consequences* would need to be clearly defined.

In exploring the weights for *Treatment efficacy* and *Treatment safety*, two members of the focus group commented that at the point that they consider drugs from a reimbursement perspective, the drug would have been approved by the European Medicines Agency or an equivalent organization (Federal Office of Public Health, Switzerland), and therefore it can be assumed that that both the efficacy and safety of the new product meet at least the expected minimum standards, and therefore these 2 criteria should not necessarily be weighted as much as the trend seemed to be.

One of the limitations of this study relates to the nature of the 2 groups. Under ideal conditions, it would be advisable to have multi-country representation in the expert focus group rather than only representation from Switzerland, thereby giving breadth to the discussion and outcomes. Nonetheless, this provided a valuable opportunity to test the concept and approach, which can now be refined and rolled out to a larger, multi-national, multi-stakeholder expert focus group. Similarly, the representation in the large group was not spread evenly by country nor profession. Since the tool is web-based, we plan to further test it by sharing it with a far wider audience, although we believe that group sessions and the discussions they elicit are far more beneficial than individual responses.

A further limitation was that the focus group included only one payer from the public sector, the sector. However, in a country where most medicines used in the community are funded through private insurance or out-of-pocket for patients, it was deemed appropriate to include a payer from the private insurance sector.

A clear omission in this study is the lack of attention given to the pricing of orphan drugs. The ever-increasing cost of orphan drugs is clearly a major cause for concern for payers and healthcare systems. The rationale for this omission was so that the focus would be only on reviewing the weighting preferences for all other criteria, and to avoid the discussion leading to criticism of orphan drug pricing, an issue that is frequently addressed in the media (62–64).

While the *Level of research undertaken* seemed to be of significant importance to the expert focus group with an average weight of 13%, the general stakeholder sample scored it an average of 7.28%. Other Product Development criteria (*Dynamic efficiency (R&D spillover), Innovation)* were considered less important by both groups. *Dynamic efficiency (R&D spillover)* was viewed as a criterion that depended on changes in the future that could only be judged retrospectively, most likely after a substantial lapse of time. The expert focus group discussion led to a heated debate on *Innovation*. The participants voiced concern for how to judge a drug's level of innovation, with some members referring to the emergence of gene therapy as potentially clouding the understanding of the criterion. The scoring system adopted in France by the Haute autorité de santé (HAS), l'amélioration du service médical rendu (ASMR; the improvement in actual benefit) (65) was considered a “good starting point” although the difference between the levels was deemed unclear. Furthermore, currently there is much debate about *real innovation* in orphan drugs because if a new treatment comes to market for a disease for which there is no treatment, then purely by comparison, the new treatment might be deemed “innovative,” whilst not necessarily providing as innovative an outcome as necessary to justify some of the high prices demanded (66). It has also been argued that the use of “salami slicing” and re-purposing of drugs merely serve to generate treatments for more indications, but without generating added innovation (67). Payers are increasingly scrutinizing the cost-benefit ratio of new orphan drugs (68).

It is of interest that *Treatment convenience* was perceived as an unimportant criterion given that if a drug has to be administered frequently in a clinical setting, it is likely to incur far more costs than a treatment that is self-administered by the patient (oral, self-inject). Furthermore, one could argue that a drug that is difficult to administer or that has to be taken multiple times daily is likely to result in poor treatment adherence (69, 70), which has a ripple of effect of suboptimal treatment outcomes, an increased risk of hospitalization (70) and wasted resources, all with negative economic outcomes.

## Conclusion

MCDA frameworks are complex models with increasing complexity as more criteria are added to the decision-making process and additional evidence generation is required (71). Some people might consider that replacing a cost-effectiveness analysis with MCDA results in added uncertainty of what the results really imply in the context of reimbursement decisions. On the one hand, HTA bodies have gained many years of experience in interpreting cost-effectiveness model outcomes. However, there is concern that cost-effectiveness does not offer a realistic method to consider an orphan drug's value-add because it does not consider the multi-dimensional factors relating to the rare disease and its treatment. Conversely, MCDA offers added insights in value assessments of orphan drugs, but at this point, there is limited experience using MCDA. This study sheds light on the important aspect of the preferences of the different criteria. All respondents agreed on the relative importance of *Treatment efficacy* and *Treatment safety*, which are captured in conventional cost-effectiveness studies, but they also expressed the view that *Disease severity, Level of research undertaken* (which could contribute to addressing uncertainty) and *Unmet need* should definitely be included in MCDA frameworks for assessing orphan drugs.

## Data Availability Statement

The datasets generated for this study are available on request to the corresponding author.

## Author Contributions

CS and MC jointly determined the overall structure of the study and aligned it with the inputs from MP and PK. OT and AV developed, tested and refined the web-based interactive MCDA tool. CS, MC, OT, and AV conducted the initial tool data capture. CS developed the protocol and questions for the interviews, recruited and managed the focus group and analyzed the data and interpretation of the findings. MP and PK reviewed analyses, interpretation and reporting for critical content. All the authors read and approved the final manuscript.

## Conflict of Interest

MP has received grants and honoraria from various pharmaceutical companies, but all unrelated to this manuscript. AV and OT were employed by the company Digital Health Outcomes for which no remuneration was made for this work. The remaining authors declare that the research was conducted in the absence of any commercial or financial relationships that could be construed as a potential conflict of interest.

## Abbreviations

AHP: Analytical hierarchy process
ASMR: Amélioration du service médical rendu
HAS: Haute autorité de santé
HTA: Health technology assessment
ISPOR: International Society for Pharmacoeconomics Outcomes Research
MAUT: Multi-attribute utility theory
MCDA: Multi-criteria decision analysis
NICE: National Institute for Health and Care Excellence
R&D: Research and development.
